# Can HIV recent infection surveillance help us better understand where primary prevention efforts should be targeted? Results of three pilots integrating a recent infection testing algorithm into routine programme activities in Kenya and Zimbabwe

**DOI:** 10.1002/jia2.25513

**Published:** 2020-06-30

**Authors:** Brian D Rice, Mariken de Wit, Susie Welty, Kathryn Risher, Frances M Cowan, Gary Murphy, Sungai T Chabata, Wanjiru Waruiru, Sitholubuhle Magutshwa, John Motoku, Daniel Kwaro, Benard Ochieng, Georges Reniers, George Rutherford

**Affiliations:** ^1^ London School of Hygiene & Tropical Medicine London UK; ^2^ University of California San Francisco USA; ^3^ Centre for Sexual Health and HIV/AIDS Research Harare Zimbabwe; ^4^ Independent consultant in HIV laboratory diagnostics London UK; ^5^ Eastern Deanery AIDS Relief Programme Nairobi Kenya; ^6^ Kenya Medical Research Institute Kisumu Kenya

**Keywords:** HIV, surveillance, recent infection, prevention, Kenya, Zimbabwe

## Abstract

**Introduction:**

Surveillance of recent HIV infections in national testing services has the potential to inform primary prevention programming activities. Focusing on procedures required to accurately determine recent infection, and the potential for recent infection surveillance to inform prevention efforts, we present the results of three independent but linked pilots of recency testing.

**Methods:**

To distinguish recently acquired HIV infection from long‐standing infection, in 2018 we applied a Recent Infection Testing Algorithm that combined a laboratory‐based Limiting Antigen Avidity Enzyme Immunoassay with clinical information (viral‐load; history of prior HIV diagnosis; antiretroviral therapy‐exposure). We explored potential misclassification of test results and analysed the characteristics of participants with recent infection. We applied the algorithm in antenatal clinics providing prevention of mother‐to‐child transmission services in Siaya County, Kenya, outreach sites serving female sex workers in Zimbabwe, and routine HIV testing and counselling facilities in Nairobi, Kenya. In Nairobi, we also conducted recency testing among partners of HIV‐positive participants.

**Results:**

In Siaya County, 2.3% (10/426) of HIV‐positive pregnant women were classified as recent. A risk factor analysis comparing women testing recent with those testing HIV‐negative found women in their first trimester were significantly more likely to test recent than those in their second or third trimester. In Zimbabwe, 10.5% (33/313) of female sex workers testing HIV‐positive through the outreach programme were classified recent. A risk factor analysis of women testing recent versus those testing HIV‐negative, found no strong evidence of an association with recent infection. In Nairobi, among 532 HIV‐positive women and men, 8.6% (46) were classified recent. Among partners of participants, almost a quarter of those who tested HIV‐positive were classified as recent (23.8%; 5/21). In all three settings, the inclusion of clinical information helped improve the positive predictive value of recent infection testing by removing cases that were likely misclassified.

**Conclusions:**

We successfully identified recently acquired infections among persons testing HIV‐positive in routine testing settings and highlight the importance of incorporating additional information to accurately classify recent infection. We identified a number of groups with a significantly higher proportion of recent infection, suggesting recent infection surveillance, when rolled‐out nationally, may help in further targeting primary prevention efforts.

## 1 INTRODUCTION

Knowing where and among whom new HIV infections are occurring is helpful in estimating HIV incidence and also, potentially, in guiding prevention programmes and evaluating their impact [1, 2, 3, 4, 5, 6, 7]. Identifying hotspots, at the population‐level, of recently acquired HIV infection could help programmes identify where and among whom primary prevention efforts such as pre‐exposure prophylaxis (PrEP) and voluntary medical male circumcision (VMMC) should be intensified. Information on recently acquired HIV may also inform primary prevention efforts at the individual level. For example prioritizing partner notification services among newly diagnosed persons who have acquired HIV recently may minimize recall bias relating to partner information [8], and assist efforts to reach a person’s most recent partners to encourage them to seek testing and preventative services.

A number of laboratory‐based assays have been developed that can identify recent HIV infections through the testing of blood specimens [9, 10]. These assays utilize specific antibody markers that evolve in the months following infection. When interpreted as part of a Recent Infection Testing Algorithm (RITA) (where laboratory test results are combined with other information to classify an HIV infection), these assays are able to distinguish recently acquired infection from long‐standing infection among persons being diagnosed with HIV [6, 10]. They have been used in national population‐based HIV impact assessment (PHIA) surveys in 12 high‐burden African countries to estimate national HIV incidence [11, 12, 13]. In 2018, the United States President's Emergency Plan for AIDS Relief (PEPFAR) called for recent infection surveillance to be implemented at scale in supported countries [14, 15]

We present the results of three independent but linked pilots of HIV recency testing in routine service‐provision settings in Kenya and Zimbabwe.

## 2 METHODS

To explore whether RITAs can be applied in routine service setting in sub‐Saharan Africa, and whether the information generated can be used to inform prevention activities, we chose a variety of routine service‐provision contexts in Kenya and Zimbabwe to conduct recency testing. These settings were as follows: antenatal clinics providing prevention of mother‐to‐child transmission (PMTCT) services in Siaya County, Kenya, a national programme for female sex workers in Zimbabwe, and HIV testing and counselling (HTC) facilities in Nairobi, Kenya.

### 2.1 Data collection and sample processing

Prior to the commencement of our pilots, all study staff underwent training on good clinical practice, ethics and the handling of confidential information as per our study protocols. Eligible participants were asked to read and sign a consent form and were probed for their understanding. For illiterate participants, study staff read the forms out and sought consent in the presence of an independent witness. Data were collected between February and November 2018.

**Figure 1:**
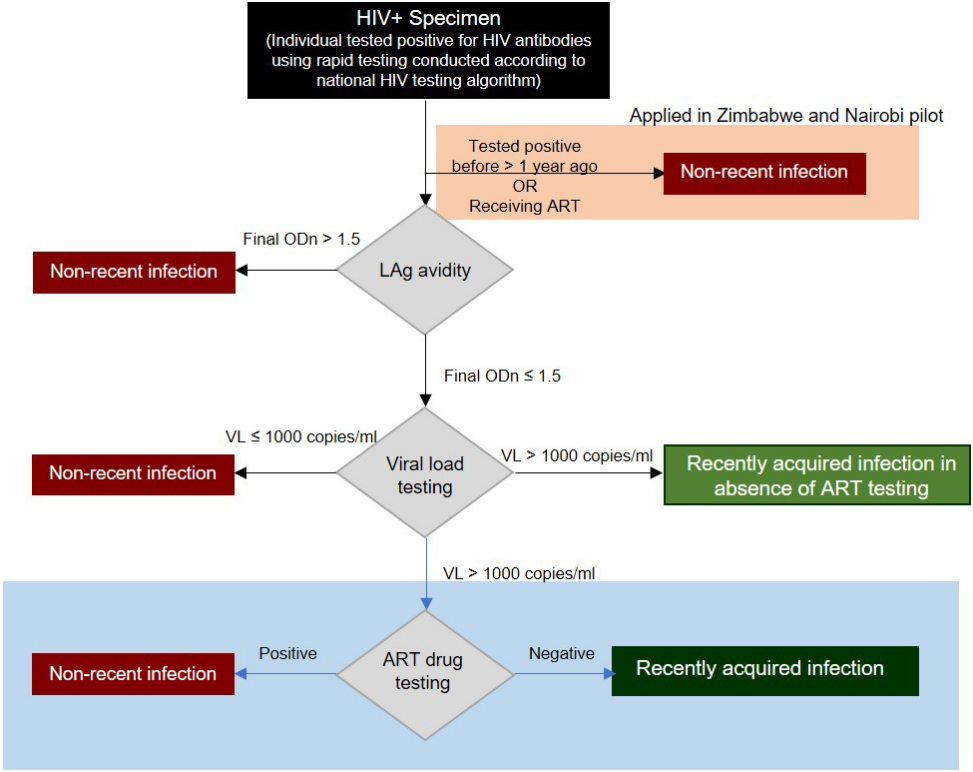
Recency testing algorithm (RITA) as applied in the three pilots.

In all three pilots, anti‐HIV‐1‐positive specimens were classified as either recent or long‐standing using a RITA that combined a LAg Avidity EIA (a single‐well avidity assay that provides a measure of antibody avidity as normalized optical density (ODn)) with information on viral‐load, history of prior HIV diagnosis and/or exposure to antiretroviral therapy (ART)) (Figure 1). This RITA gives an indication as to whether or not a person being diagnosed with HIV is likely to have been infected within the last four to six months.

The Maxim HIV‐1 LAg‐Avidity EIA Dried Blood Spot (DBS) Kit was used in Nairobi on DBS samples, with the Maxim HIV‐1 LAg‐Avidity EIA being used in Siaya County and Zimbabwe on plasma samples. As per manufacturer guidance, LAg tests with an initial ODn value ≤ 2.0 were retested in triplicate from a fresh dilution of the specimen to confirm the result, and confirmatory anti‐HIV serology was performed on specimens with an ODn value < 0.4. Viral‐load was measured using the Abbott m2000, Roche Cobas Ampliprep/Cobas Taqman or similar automated platform, according to manufacturers’ instructions. Internal quality control checks were run according to manufacturers’ instructions. Persons with a final ODn ≤ 1.5 and a viral‐load> 1000 copies/ml were classified as recent infection positive in the absence of ART testing.

As the inclusion of information on ART‐exposure could improve RITA performance through lowering false‐recent misclassification [4, 16], samples assessed as recent in Nairobi were sent to the Pharmacokinetic Laboratory at the University of Cape Town to test for the presence of ART metabolites in the blood (metabolites, including Lopinavir, Ritonavir, Nevirapine, Efavirenz, Indinavir, Saquinavir, Zidovudine, Lamivudine and Stavudine, were quantified by a robust simultaneous liquid chromatography/tandem mass spectrometry method)[17, 18].

In Siaya County, recent samples were linked to a woman’s antenatal clinic record to explore ART‐exposure, and were also linked to their antenatal clinic and Health and Demographic Surveillance Site (HDSS) record to explore testing history for prevalent infection. Evidence of ART‐exposure or previous HIV‐positive test would potentially result in a recent infection being reclassified as long‐standing. Table 1 summarizes our recruitment and testing approach per pilot.

**Table 1:** Three pilots of HIV recent infection testing in routine service settings

Siaya County, Kenya	Nairobi, Kenya	Sisters with a Voice, Zimbabwe
**Setting** Estimated HIV prevalence of 21% in 2017 [26]. Fertility rate> five children per woman, and almost all women (94%) access antenatal care at some point during pregnancy [27].	**Setting** Estimated HIV prevalence of 12% in population served by participating clinics in Eastern Nairobi [28].	**Setting** Across participating facilities, prevalence of HIV among FSW is on average 58% [20, 29, 30]. HIV incidence rates are poorly understood, but may be as high as 10% per year [31].
**Collaborative partner** Kenya Medical Research Institute (KEMRI) and the KEMRI/CDC Siaya HDSS	**Collaborative partner** Eastern Deanery AIDS Relief Programme (EDARP)	**Collaborative partner** Centre for Sexual Health and HIV AIDS Research Zimbabwe (CeSHHAR‐Zimbabwe)
**Study population** Pregnant women seeking antenatal care in fourteen medical facilities	**Study population** Clients attending any of the fourteen EDARP HTC facilities	**Study population** FSW attending one of six static facilities of the Sisters with a Voice Programme that provide a range of services including testing and referral to government ART services [31].
**Study period** February – November 2018	**Study period** March – November 2018	**Study period** June – November 2018
**Assay** Maxim HIV‐1 LAg‐Avidity EIA venous blood	**Assay** Maxim HIV‐1 LAg‐Avidity EIA Dried Blood Spot	**Assay** Maxim HIV‐1 LAg‐Avidity EIA venous blood
**Inclusion criteria** Women aged 13 or older seeking antenatal care in one of the selected medical facilities in Siaya County Provides informed consent Received an HIV‐positive test result	**Inclusion criteria** Aged 18 or older Unknown HIV status prior to visit Attending an EDARP HTC facility Willing and able to provide informed consent Received an HIV‐positive test result, or presumptive positive	**Inclusion criteria** FSW aged 18 or older Provides informed consent Received an HIV‐positive test result
	**Exclusion criteria** Indeterminate HIV result Not willing to enrol on follow‐up at facility Taking pre‐exposure prophylaxis	**Exclusion criteria** Indeterminate HIV result Prior history of testing HIV‐positive (>1 year ago) On ART
**Specimen collection and testing** Study nurse or laboratory phlebotomist drew a maximum of 10ml of venous blood Samples packed and transferred to KEMRI‐Centre for Global Health Research HIV Research Laboratory in Kisumu on a daily basis where they were tested (or stored for testing)	**Specimen collection and testing** Study nurse drew 6mL of venous blood collected in an ethylenediaminetetraacetic (EDTA) tube and a pipette was used to dispense venous blood on two Whatman^TM^ 903 Snap‐Apart Cards with 5 dried blood spots (DBS) of 70 µL each, for a total of 10 filled spots per participant	**Specimen collection and testing** Study nurse drew venous blood (where study nurse not available, then a clinic nurse drew blood) Samples packed and transferred to laboratory in Harare within 36 hours and stored at −20C or below for testing

Inclusion and exclusion criteria for each pilot reflect the routine practice of the facilities within which recruitment was conducted. FSW, Female Sex Workers; HDSS, Health and Demographic Surveillance System; HTC, HIV Testing and Counselling; EIA, Enzyme‐linked Immunosorbent Assay.

For all three pilots we collected information on participants’ sex, age, marital status and testing facility. In Zimbabwe and Nairobi, level of education was also collected. In addition, number of pregnancies and pregnancy trimester was collected in Siaya County, and employment status, HIV testing history and pregnancy status was collected in Nairobi.

### 2.2 Statistical analysis

We developed a flowchart to show the flow of participants and sample testing for all three pilots together, starting with all those presenting for testing, and ending with final classification of recent and long‐standing HIV infection. We present the number of people testing recent prior and subsequent to viral‐load testing and ART investigations. In relation to ART‐exposure, we detail each misclassification case.

Using the Siaya county and, separately, the Zimbabwe data we describe the characteristics of those testing HIV‐positive and classified as recent. To look for risk factors for recent HIV infection, we applied logistic regression where women testing HIV‐positive and recent were compared with those testing HIV‐negative. In Siaya County, due to a small number of recent cases, it was not possible to apply logistic regression adjusting for multiple variables.

Using the Nairobi HTC data, we describe the characteristics of HIV‐positive women and men with recent and long‐standing infection. We applied logistic regression to compare the characteristics of those with recent infection with those with long‐standing infection (it was not possible to compare to persons testing HIV‐negative as these data were not available). To account for facility clustering, a generalized estimated equation (GEE) model that includes age at diagnosis, gender, and HIV testing history was applied. In Nairobi, participants confirmed to be HIV‐positive, were counselled on index‐testing and asked to bring their sexual partners to the facility for HIV and recent infection testing. We describe the characteristics of these partners.

Due to small cell counts, and to avoid deductive disclosure, testing facility was either anonymized or combined in our analysis. Percentages are presented as among persons for whom information was available, confidence intervals are presented at the 95% level, and Wald and likelihood‐ratio tests were applied for logistic regression. In Zimbabwe (only pilot for which the necessary data were available), we assessed recent infection clustering by sample collection date and testing facility. STATA15 (Stata Corp, College Station, TX) was used for analyses.

### 2.3 Ethical approval

Local approval was provided by KEMRI Scientific Ethics Review Unit (SERU application 3589) and London School of Hygiene & Tropical Medicine (LSHTM) (reference number 14458) for the pilot in Kisumu, by the ethical committee of Medical Research Council of Zimbabwe for the Zimbabwe pilot and by Kenyatta National Hospital‐University of Nairobi Ethical Review Board for the pilot in Nairobi. Ethical approval was also obtained at the LSHTM for the Zimbabwe pilot (reference number 14542) and Nairobi pilot (reference number 14585).

## 3 RESULTS

### 3.1 Siaya County, Kenya: antenatal clinics providing PMTCT services

**Figure 2:**
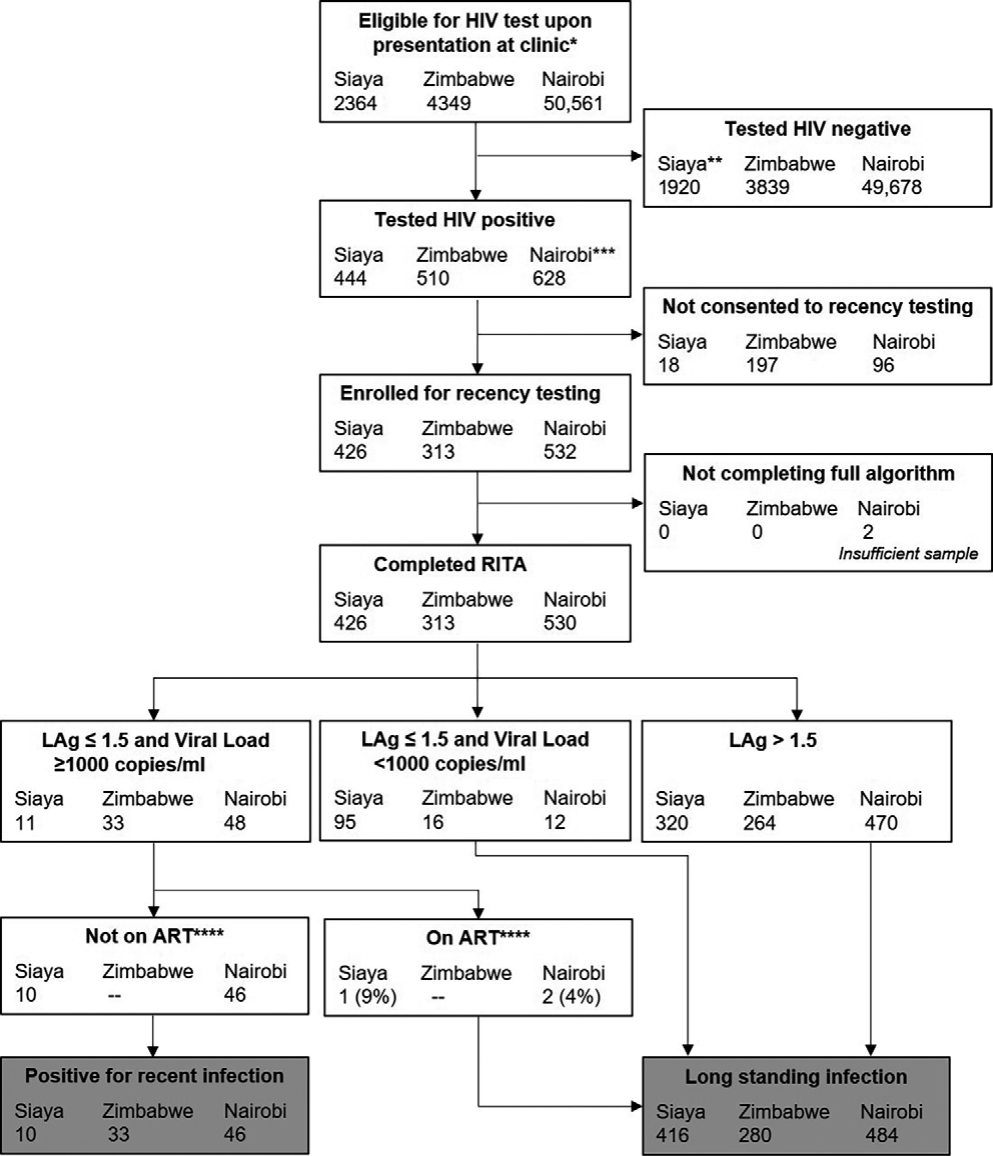
Recruitment and testing flowchart for the three pilots. ^*^Eligibility criteria: providing consent for HIV testing & in Zimbabwe: not having been tested in previous three months or taking ART. ^**^1914 tested HIV‐negative, six had unknown HIV status. ^***^883 tested HIV‐positive, but 255 of these tested HIV+ before. ^****^In Siaya County, ART status was determined using clinic records; in Nairobi, ART status was determined using ARV metabolite testing; in Zimbabwe, ART status was not determined. RITA, recency testing algorithm

Over the study period, 2409 eligible women presented at participating antenatal clinics, of whom 2364 (98.1%) consented to participate in the study (Figure 2). Of these women, 1806 (76.4%) were under 30 years of age, 1792 (75.8%) were married and 1157 (48.9%) had experienced three or more pregnancies. In total, 444 (18.8%) women tested HIV‐positive, of whom 426 (95.6%) had a valid LAg and viral‐load test (18 women did not consent to recency testing). Among these 426 women, 106 (24.9%) tested recent prior to viral‐load being considered, with 11 (2.6%) testing recent with a viral‐load> 1000 copies/mL.

Of the 11 women classified as recent based on their LAg and viral‐load tests, one was reclassified as long‐standing when clinical documentation of treatment was also considered (recency misclassification of 9.1% (1/11)). For this woman there was clinical documentation of her having initiated treatment almost four years prior to their sample collection date within the study. Of the remaining ten women, one initiated treatment 78 days prior to her study sample draw date. As her first known HIV‐positive date was on the same date as ART initiation, she remained classified as recent. Another woman initiated ART three days prior to her study sample draw, and again remained classified as recent. Of the remaining eight women, four initiated treatment on the same day as their study sample draw, three initiated within 16 days of their sample draw, and one did not have documented date of ART initiation. Exploration of the women’s HIV testing history for prevalent infection from the HDSS and ANC record, provided no further evidence of misclassification. In total 2.3% (10/426) of women were classified recent.

A total of 10 women classified as recent were identified at seven of the 14 clinics and had sample collection dates evenly distributed across the study period. Nine (90%) of the 10 women were under 30 years of age, and half (55.6%; 5/9 (1 missing record)) were in their first trimester of pregnancy.

Comparing HIV‐positive women testing recent with women testing HIV‐negative found women in their first trimester to have nearly a ten times increased odds of testing recent compared to those in their second or third trimester (Table 2). There were no notable differences in age, marital status, study facility, or number of pregnancies between HIV‐negative women and HIV‐positive women testing recent.

**Table 2:** Characterization of HIV and recent infection among antenatal clinic attendees in Siaya County testing for HIV

Characteristics	HIV positivity	Risk factor analysis for recent infection		
	n/N^a^ (%)	n/N^b^ (%)	Crude OR (95% CI)	*p*‐value
Age (years)				
<20	30/503 (6.0)	2/475 (0.4)	0.74 (0.10 to 3.8)	0.72
20 to 24	100/794 (12.6)	4/698 (0.6)	1	—
25+	314/1060 (29.6)	4/750 (0.5)	0.93 (0.22 to 3.9)	0.92
Study site				
1^c^	102/522 (19.5)	3/423 (0.7)	1.5 (0.33 to 5.5)	0.54
Other	342/1836 (18.6)	7/1501 (0.5)	1	
Marital status				
Married	364/1787 (20.4)	7/1430 (0.5)	1	
Single	40/500 (8.0)	2/462 (0.4)	0.88 (0.13 to 3.7)	0.88
Separated/ divorced/ widowed	39/69 (56.5)	1/31 (3.2)	6.8 (0.36 to 39.8)	0.08
Trimester				
1^st^	74/289 (25.6)	5/215 (2.3)	9.6 (2.5 to 39.2)	<0.001
2nd & 3rd	347/2005 (17.3)	4/1658 (0.2)	1	—
Pregnancies				
1	39/663 (5.9)	3/627 (0.5)	1	
2	91/519 (17.5)	5/433 (1.2)	2.4 (0.59 to 11.9)	0.22
3+	302/1157 (26.1)	2/857 (0.2)	0.49 (0.06 to 2.9)	0.43

a n = testing HIV‐positive; N = testing for HIV

b n = testing recent; N = testing HIV‐negative + testing recent

c Testing facility 1is the largest facility by patient volume

### 3.2 Zimbabwe: national programme for female sex workers

In Zimbabwe, 9138 women presented at one of the six participating facilities, of whom 4349 (47.6%) were tested for HIV (routinely, women who have tested for HIV within the past three months, or who report ART use, are not offered an HIV test). Of these 4349 women, 511 (11.7%) tested HIV‐positive, and of these, 313 (61.3%) agreed to have a sample taken for viral‐load and recent infection testing. Almost half (141; 46.7%) of these women were aged between 25 and 34 years, and the majority attained a secondary school education (247; 81.8%) and/or were separated or divorced (177; 58.6%).

Among the 313 women who tested HIV positive, and for whom both a viral‐load and a recent infection test was available, 15.7% (49/313) tested recent based on their LAg test result alone. Based on their LAg and viral‐load (>1000 copies/mL), 33 (10.5%) of these women were classified as recent (Figure 2). Among the 313 women testing HIV‐positive, by age, those aged 18 or 19 years had the highest percentage of recent infection (5/23; 21.7%). By education, HIV‐positive women for whom secondary education was their highest attainment were most likely to test recent (31/246; 12.6%), whereas by marital status, those who were single or never married presented with the highest percentage of recent infection (17/87; 19.5%).

Table 3 characterizes HIV positivity among the 4349 women tested for HIV, and also presents a risk factor analysis of recent infection, comparing HIV‐positive women with recent infection to HIV‐negative women, adjusting for age and study facility. There was no strong evidence of an association between having a recent infection and any of the variables.

**Table 3:** Characterization of HIV and recent infection among female sex workers testing for HIV in Zimbabwe

	HIV positivity	Risk factor analysis for recent infection				
	n/N^a^ (%)	n/N^b^ (%)	Crude OR (95% CI)	*p*‐value	Adjusted OR^c^ (95% CI)	*p*‐value
Age (years)				0.37		0.3
18 to 19	39/405 (9.6)	5/372 (1.4)	1		1	
20 to 24	143/1309 (10.9)	13/1180 (1.1)	0.82 (0.29 to 2.31)		0.79 (0.28 to 2.25)	
25 to 34	232/1679 (13.8)	13/1460 (0.9)	0.66 (0.23 to 1.86)		0.60 (0.21 to 1.74)	
35+	78/676 (11.5)	2/600 (0.3)	0.24 (0.05 to 1.27)		0.22 (0.04 to 1.16)	
Study site				0.18		0.12
1	89/744 (12.0)	6/661 (0.9)	1		1	
2	57/414 (13.8)	1/358 (0.3)	0.31 (0.04 to 2.55)		0.33 (0.04 to 2.79)	
3	228/1518 (15.0)	11/1302 (0.9)	0.93 (0.34 to 2.53)		1.04 (0.38 to 2.87)	
4	37/357 (10.4)	5/325 (1.5)	1.71 (0.52 to 5.63)		2.08 (0.62 to 6.94)	
5	61/477 (12.8)	7/423 (1.7)	1.84 (0.61 to 5.50)		2.08 (0.69 to 6.28)	
6	39/839 (4.7)	3/803 (0.4)	0.41 (0.10 to 1.64)		0.42 (0.10 to 1.69)	
Education				0.15		0.15
Primary or less	87/635 (13.7)	2/550 (0.4)	1		1	
Secondary or higher	400/3374 (11.9)	31/3005 (1.0)	2.87 (0.68 to 12.01)		2.89 (0.68 to 12.24)	
Marital status				0.12		0.07
Single/ never married	148/1341 (11.0)	17/1210 (1.4)	1		1	
Married/ living together as if married	30/149 (20.1)	2/121 (1.7)	1.18 (0.27 to 5.17)		1.82 (0.39 to 8.45)	
Divorced/ separated	290/2372 (12.2)	13/2098 (0.6)	0.44 (0.21 to 0.90)		0.38 (0.17 to 0.84)	
Widowed	24/188 (12.8)	1/165 (0.6)	0.43 (0.06 to 3.24)		0.50 (0.06 to 4.18)	

a n = testing HIV‐positive (of the 511 women testing HIV positive, regardless of viral‐load and recency test); N = testing for HIV.

b n = testing recent; N = testing HIV‐negative + testing recent.

c Adjusted for age and study site. OR, odds ratio; CI, confidence interval.

A visual assessment of the data suggested some clustering by sample collection date and testing facility. On one day during week five of recruitment, six women tested positive for recent infection. Three of these women tested at the same facility, and three were aged between 20 and 24 years. A statistical analysis of clustering over time (logistic regression with sample collection date as covariate) found no evidence of an association between recency test results and week (*p* = 0.74) or month (*p* = 0.21).

### 3.3 Nairobi, Kenya: routine HIV testing and counselling clinics

In Nairobi, 50,561 eligible women and men presented at one of the fourteen participating facilities. Of these, 883 (1.75%) tested HIV‐positive, of whom 255 (28.9%) were subsequently found on enquiry (self‐reported test history) to have tested HIV‐positive before and were therefore ineligible for recency testing (Figure 2). In total, 532 (84.7%; 628) of those eligible consented to test for recent infection. Among these, 316 (59.4%) were female, of whom 57 (18%) were pregnant. The majority of participants (64.1%; 341) had previously tested for HIV, with a third (33.6%; 179) having tested in the past 12 months.

Two of the 532 people consenting to participate were subsequently found to have insufficient sample to test. Of the remaining 530 people testing for recent infection and viral‐load, 60 (11.3%) tested recent based on the LAg test result alone, with 48 (9.1%) testing recent with a viral‐load> 1000 copies/ml (Table 4). ART metabolite testing identified one woman and one man among these 48 to have been wrongly classified (misclassification of 4.2%). In total, 8.7% (46/530) of people were classified as recent. Among women this percentage was 12.4% (39/315) and among men 3.3% (7/215).

**Table 4:** Characterization of recent and long‐standing infection among women and men newly testing HIV‐positive in HIV testing and counselling facilities in Nairobi

Characteristics^a^	Recent		Long‐standing			Recency^b^
N = 530	N = 46	%		N = 484	%	%
Sex						
Male	7	15.2		208	43.0	3.3
Female	39	84.8		276	57.0	12.4
Age (years)						
15 to 19	5	10.9		13	2.7	27.8
20 to 24	16	34.8		79	16.3	16.8
25 to 29	13	28.3		102	21.1	11.3
30 to 34	7	15.2		100	20.7	6.5
35 to 39	2	4.3		80	16.5	2.4
40+	3	6.5		110	22.7	2.7
Testing facility						
1^c^	9	19.6		36	7.5	20.0
Other	37	80.4		447	92.5	7.6
Marital status						
Single	15	32.6		97	20.0	13.4
Married/co‐habiting	24	52.2		269	55.6	8.2
Separated	6	13.0		74	15.3	7.5
Divorced	0	0.0		10	2.1	0.0
Widowed	1	2.2		31	6.4	3.1
Unknown	0	0.0		3	0.6	0.0
Highest level of education						
None	2	4.3		7	1.4	22.2
Primary	16	34.8		251	51.9	6.0
Secondary	21	45.7		176	36.4	10.7
Tertiary	7	15.2		48	9.9	12.7
Unknown	0	0.0		2	0.4	0.0
Employment status						
Employed	28	60.9		347	71.7	7.5
Unemployed	18	39.1		135	27.9	11.8
Unknown	0	0.0		2	0.4	0.0
Ever tested for HIV						
Yes	35	76.1		305	63.0	10.3
No	11	23.9		179	37.0	5.8
Tested for HIV in last 12 months						
Yes	25	54.3		153	31.6	14.0
No	21	45.7		329	68.0	6.0
Unknown	0	0.0		2	0.4	0.0
Pregnancy status (n = 316)						
Pregnant	10	25.6		47	17.0	17.5
Not‐pregnant	29	74.4		229	83.0	11.2

a Excludes two participants for whom there was insufficient sample to test

b Calculated as recent infection/ newly testing HIV‐positive (recent & long‐standing infections); the denominator only includes new HIV‐positives as repeat testers were excluded

c Testing facility 1 is the largest facility by catchment area and patient volume

Around half (45.7%; n = 21) of the 46 people newly testing HIV‐positive who also tested recent were aged under 25 years and the proportion with a recent infection declined with increasing age, see Table 4. Just over half of those testing recent were married or co‐habiting (52.2%; n = 24), and had tested HIV‐negative in the past 12 months (54.3%; n = 25). The percentage of people newly testing HIV‐positive who had previously tested for HIV during the past 12 months was higher among those classified as recent (54.3%; n = 25) than among those classified as having long‐standing infection (31.6%; 153/484).

Among people newly testing HIV‐positive (recent and long‐standing infection), being a woman, being under 25 years of age, having tested for HIV in the last 12 months, and presenting at the facility with the largest catchment area and largest corresponding patient volume were shown to be individually predictive of recent infection (Table 4). Testing for interactions indicated an interaction between age at diagnosis and gender (Table 5). Young women (15 to 29 years old) had 3.85 times the odds of recent infection than men in the same age group. Participants reporting having tested for HIV in the past 12 months had 1.72 times the odds of recent infection compared to those reporting having last tested more than 12 months ago.

**Table 5:** Predictors of recent infection, disaggregated by gender, among women and men newly testing HIV‐positive in HIV testing and counselling facilities in Nairobi

	Male			Female		
	N	aOR (95%CI)	*p*‐value	N	aOR (95%CI)	*p*‐value
Aged 15 to 29 years	58	1	‐	171	1	‐
Aged 30+ years	158	0.99 [0.94, 1.06]	0.87	145	0.23 [0.16, 0.32]	<0.01
Tested over 12 months ago	162	1	‐	189	1	‐
Tested within last 12 months	53	1.05 [1.01, 1.08]	0.01	126	2.12 [1.83, 2.45]	<0.01

Following an HIV‐positive test result and counselling, 144 (27.1%) of participants subsequently brought a sexual partner to the clinic for HIV testing. Of these 144, two brought in two partners, making the total 146. Among the 146 sexual partners of index cases testing for HIV, 61 (41.8%) tested positive for HIV. Among these 61 HIV‐positive partners, 21 subsequently enrolled in the pilot, of whom five (5/21; 23.8%) were classified as recent. The percentage of partners testing HIV‐positive, and being classified as recent, were significantly higher than the corresponding percentages among all non‐partner (i.e. the recruitment figures presented above less the 146 partners) participants (*p* < 0.001 & *p* = 0.019 respectively).

## 4 DISCUSSION

We successfully conducted three independent but linked pilots in routine programme setting in Kenya and Zimbabwe to identify people with recently acquired HIV infection. Among HIV‐positive participants, we report 2.3% of pregnant women in Siaya County, 10.5% of FSW in Zimbabwe and 12.4% of women and 3.3% of men attending HTC facilities in Nairobi to have been diagnosed with recent infection. Among partners of participants in Nairobi, the percentage was 23.8%.

In Siaya County we found women in their first trimester to be significantly more likely to test recent compared to those in their second or third trimester. While inference with small numbers is challenging, this finding may relate to increased risk of HIV infection during unprotected sex leading to pregnancy, and/or may be due to lowered coital frequency among women later in pregnancy. The former of these two potential explanations would support the targeting of women trying to get pregnant, particularly those in sero‐discordant couples, with interventions such as PrEP and partner testing. In Kenya, the offering of HTC to the partners of persons diagnosed with HIV is already encouraged [19].

Among FSW in Zimbabwe, there was no strong evidence of associations with testing recent when comparing HIV‐positive women with recent infection to HIV‐negative women. One likely reason for this is the relatively small number of women found to have a recent infection. Despite a lack of association, and despite FSW in Zimbabwe being a known high‐risk group [20, 21], we would argue that ongoing efforts to differentiate prevention needs among FSW are applicable. These efforts should include the expanded roll‐out of recency testing at venues utilized by FSW and, where possible, through the collection of more detailed information on previous HIV test history.

In Nairobi, we found young women newly testing HIV‐positive were significantly more likely to test recent than their male counterparts. The observed difference by sex is probably due to women presenting earlier in their course of infection than men. Among those newly testing HIV‐positive, women, as compared to men, were younger at age of HIV diagnosis, and were more likely to have ever tested and to have tested within the last 12 months. We were unable to investigate why women may present earlier than men. We also found participants newly testing HIV‐positive who had tested for HIV in the past 12 months were significantly more likely to test recent than those having tested over a year ago. This probably reflects that people who are HIV‐negative that test more frequently (potentially due to engaging more frequently in risk behaviour) will test recent when first diagnosed with HIV. Interestingly, we found significantly higher yields of HIV and recent infection among partner, as compared to non‐partner, participants in Nairobi. Although based on small numbers, these results provide support for recency test results among index cases informing partner testing strategies [15].

In two of our three pilots there was some suggestion of clustering by sample collection date or testing facility. In Zimbabwe, six of the 33 women testing recent did so on the same day, with three also testing at the same facility. In Nairobi, the facility with the largest catchment area and patient volume presented with a significantly higher percentage of recent infections than at smaller facilities. Although we only present suggestive evidence, information on recent infection clustering in time and space could be used to target prevention efforts in specific geographical areas and specific populations; for example mobilizing outreach teams to promote HIV testing or deliver PrEP.

Several studies have explored recent infection testing in sub‐Saharan Africa. A number of these studies conducted recency testing as a means to estimate HIV incidence rather than an end in itself [22, 23]. However, a couple of studies provide details on recency testing and present recency percentages similar to those we report. Among participants aged 15 to 49 years providing DBS samples as part of the 2012 national population‐based household surveys in Kenya and South Africa, 4.5% (21/470) and 3.3% (73/2,202) tested recent respectively [4]. The authors of this study conclude that information on viral‐load and ART‐exposure in their RITA potentially improved the predictive value of the RITA [4]. Another study tested the DBS stored samples of people aged 15 to 64 years participating in the 2007 Kenya AIDS Indicator Survey for recent infection. Among these participants, 6.2% (64/1,025) were classified as recent [3]. Compared to persons testing HIV‐negative, factors associated with recent infection in this population included being widowed or currently married (compared to being never married), having had two or more sexual partners in the last year, not using a condom at last sex in the past year, and reporting a sexually transmitted infection diagnosis or symptoms in the past year [3].

There were a number of limitations to our study. In Siaya county and Zimbabwe recency testing was conducted on venous blood samples whereas in Nairobi DBS was used. As we did not compare the performance of these two methods, results across the three pilots should be compared with caution. We planned to conduct ART metabolite testing in all three pilots. Due to challenges in attaining import and export licences to transport samples to the University of Cape Town we were only successful in carrying out ART testing in our Nairobi pilot. In Zimbabwe, routinely applied criteria for conducting an HIV test reduced the number of women eligible for inclusion in our study, thereby potentially reducing the number of women found to be HIV‐positive and recent. Furthermore, the fairly low participation rate in recency testing (61.3%) among the women testing HIV‐positive ensures our results should be interpreted with caution, given the possibility for selection bias. In Nairobi, we were unable to attain detailed information on people testing HIV‐negative. Therefore, in contrast to Siaya County and Zimbabwe, we compare persons testing recent with long‐standing infection rather than HIV‐negativity.

For programmes to include recency testing as part of routine HIV service delivery for the purpose of identifying priority populations for primary prevention efforts (the first step of a unifying HIV prevention cascade framework described by Schaefer *et al*) [24], there are a number of considerations. Programmes can anticipate additional costs resulting from test assays and logistics related to sample handling [25], needing to make modifications to client flows (e.g. to draw additional amounts of blood for laboratory‐based testing), and providing additional training to healthcare workers. The collection, transportation, storage and testing of samples (DBS and plasma) will need to be closely monitored to ensure assay manufacturer’s instructions for use are being followed, and testing laboratories will require training in the performance of the assay and should partake in an external quality‐assurance scheme. To guarantee the inclusion of ART metabolite testing in the interpretation of LAg test results, the process for transporting samples outside of country should be reviewed prior to commencement of testing, and simpler assays should be developed so that blood samples may be tested at in‐country laboratory facilities. Finally, programmes will need to consider potential prevention interventions resulting from recent infections, such as partner testing, PrEP delivery and community prevention initiatives.

## 5 CONCLUSIONS

At a time when the national surveillance of recent infection is being promoted across sub‐Saharan Africa [14, 15], we show that integrating recency testing into routine programme activities in Kenya and Zimbabwe is feasible. In identifying recently acquired infections among persons testing HIV‐positive, we highlight the importance of incorporating information on viral‐loads and ART to accurately classify recent infection. Having identified a number of groups with a significantly higher proportion of recent infection, we highlight how recency surveillance may help us in further targeting primary prevention efforts. The identification of hotspots of transmission and characteristics associated with new infection, even among high‐incidence populations, could inform where and among whom primary prevention efforts should be strengthened.

## COMPETING INTERESTS

We, the authors, have no conflicts of interest to declare.

## AUTHORS’ CONTRIBUTION

BR wrote the paper and conceived, organized and led the study. MdW, SW, KR and SC analysed the data. WW, SM, JM, DK and BO coordinated the study. FC, GReniers and GRutherford implemented the work. GM supported the laboratory analyses. All authors have read and approved the final manuscript.
